# Rapid, low cost prototyping of transdermal devices for personal healthcare monitoring

**DOI:** 10.1016/j.sbsr.2016.10.004

**Published:** 2017-04

**Authors:** Sanjiv Sharma, Anwer Saeed, Christopher Johnson, Nikolaj Gadegaard, Anthony EG Cass

**Affiliations:** a Department of Chemistry & Institute of Biomedical Engineering, Exhibition Road, South Kensington Campus, Imperial College London, SW7 2AZ, UK; b School of Engineering, University of Glasgow, Glasgow G12 8LT, UK

**Keywords:** Microneedles, Minimally invasive sensors, Continuous glucose monitoring (CGM), Continuous lactate monitoring (CLM), Interstitial therapeutic drug monitoring (iTDM)

## Abstract

The next generation of devices for personal healthcare monitoring will comprise molecular sensors to monitor analytes of interest in the skin compartment. Transdermal devices based on microneedles offer an excellent opportunity to explore the dynamics of molecular markers in the interstitial fluid, however good acceptability of these next generation devices will require several technical problems associated with current commercially available wearable sensors to be overcome. These particularly include reliability, comfort and cost. An essential pre-requisite for transdermal molecular sensing devices is that they can be fabricated using scalable technologies which are cost effective.

We present here a minimally invasive microneedle array as a continuous monitoring platform technology. Method for scalable fabrication of these structures is presented. The microneedle arrays were characterised mechanically and were shown to penetrate human skin under moderate thumb pressure. They were then functionalised and evaluated as glucose, lactate and theophylline biosensors. The results suggest that this technology can be employed in the measurement of metabolites, therapeutic drugs and biomarkers and could have an important role to play in the management of chronic diseases.

## Highlights

• Microneedle structure based minimally invasive, transdermal sensors for measuring metabolites and drugs in the dermal compartment
• These microneedle structures are fabricated by injection molding technology that has potential for mass production
• Microneedle structures are mechanically robust against transverse and axial forces
• Microneedle structures penetrate human skin and sit in the dermal interstitial space under moderate thumb pressure (< 10 N)
• Microneedle structures can be easily modified to glucose, lactate and theophylline biosensor devices

## 1 Introduction

The current generation of wearable devices for personal wellness monitoring such as the Fitbit (Fitbit), UP3 (Jawbone) and SIM band (Samsung) comprises a variety of physical sensors that measure parameters such as movement, heart rate, electrocardiogram (ECG), galvanic skin response, bioimpedance, and skin temperature. The next generation of wearable smart devices will also incorporate non-invasive/minimally invasive molecular sensors. The main challenges anticipated for these smart devices would be similar to the ones faced currently by the physical sensors particularly in terms of reliability, cost and the ethical issues associated with ownership and use of continuous bio-signal obtained in real time.

Transdermal devices consisting of microstructures such as hollow [25], swellable [1], [27], coated [8], [13], [26], and solid microneedles [2], [6], [14], [24], have been used mainly for vaccine [25], and drug delivery [12], [22] but there is a rapidly growing interest in using them for molecular sensing [4], [7], [15], [17], [19], [23]. The interstitial fluid (ISF) in the skin compartment exhibits a dynamic exchange of small to medium sized molecules with capillary blood thereby offering access to analytes with minimal trauma and in a continuous manner, something that could be potentially of great clinical utility. The main application where minimally invasive, continuous measurement is of immense value is in continuous glucose monitoring for diabetes. However with suitable devices this could also be extended to lactate monitoring in athletes for performance training, interstitial therapeutic drug monitoring as well as lifestyle related applications such as help in smoking cessation.

Interstitial fluid has been extensively explored for metabolites such as glucose and lactate [3], [18]. Continuous glucose monitoring (CGM) has been most intensively studied and there is good clinical evidence that effective CGM in Type 1 diabetes leads both to a reduced frequency of hypoglycaemic episodes and lowered HbA_1c_ [21].

Minimally invasive, continuous monitoring of lactate in the interstitial fluid could also offer a more informative measure of this metabolite that would allow athletes to monitor it during their training rather than taking a single (venous blood) measurement at the end [9].

The ISF microenvironment also offers a compartment in which to measure the dynamics of therapeutic drugs and biomarkers during treatment. There are reports suggesting that there are as many as 80 molecular markers in the skin compartment that show good correlation with venous blood [20]. A recent study in animals has shown a good correlation between the concentration and dynamics of many drugs in ISF and venous blood [11]. Therapeutic drug monitoring in the interstitial fluid using minimally invasive devices for continuous or periodical monitoring of drugs with narrow therapeutic index offers a potential route to personalisation of medication regimes.

Despite the promise of minimally invasive continuous monitoring, experience with CGM using some of the approved commercial devices such as those from Medtronic (Enlite), Dexcom (G5) and Abbott (FreeStyle Libre) has revealed that their uptake is still < 10% despite nearly two and half decades of research and development [10], [16]. This can be attributed mainly to poor accuracy and precision (giving rise to many false alarms) and high costs of device manufacture. Current CGM devices are mostly subcutaneously implanted and hence the emphasis has been on long term performance. Our approach has been to use a minimally invasive microneedle array format where the sensors can be readily and painlessly inserted and where the cost is sufficiently low that daily replacement is feasible. This could give all the proven benefits of continuous monitoring at a price comparable to the current single measurement strips. We report here on methods used for fabrication of such microneedle arrays.

To achieve the low cost and scalable manufacture injection molding of polycarbonate yielded 300-microneedle structures/hour. Mechanical characterisation of these structures is presented and their functionalisation to produce glucose, lactate and theophylline biosensors is described. The *in vitro* characterisation of these, as well as *in vivo* studies on human skin illustrates their performance.

## 2 Experimental section

### 2.1 Materials

Cu—W, aluminum blocks and hardened stainless steel stamps were obtained from Erodex, UK respectively. Phenol was obtained from Sigma-Aldrich, UK. Glucose oxidase (Glucose Oxidase HPS300, Sekisui Diagnostics (U.K.), Ltd., Units, Activity 239 U/mg powder @ 25 °C, source *Ex Aspergillus niger*) and lactate oxidase (Lactate Oxidase II, Sekisui Diagnostics (U.K.), Ltd., Units, Activity 41 U/mg powder @ 37 °C, source *Ex Aerococcus vinidans*) were employed as the enzymes for glucose and lactate sensors respectively. Xanthine oxidase (Xanthine Oxidase microbial, Sigma Aldrich U.K., > 7 U/mg powder) was used for the theophylline sensor.

### 2.2 Fabrication of the base microneedle array

Copper–tungsten and stainless steel masters, fabricated using electric discharge milling (EDM), were then used as electrodes for spark erosion of aluminum blocks to create molds. The spark erosion was repeated up to six times. In one instance, the molds were washed with a pressurized jet to remove the eroded debris. In another example, the aluminum mold created by EDM was pressed against a hardened stainless steel master. For the injection molding, a hydraulic injection-molding machine (Victory 28, Engel) was used to manufacture the samples. The molded parts were manufactured in polycarbonate (Makrolon OD2015) which was dried at 110 °C for 24 h under vacuum prior to use. The EDM prepared aluminum master inlay was inserted into the tool which was designed to produce parts measuring ≈ 25 × 25 × 2 mm or 25 × 25 × 1 mm. For the manufacturing of the part, we applied the following conditions to the injection molding process: *T*_m_ = 270 °C (PC melt temperature), *T*_w_ = 80 °C (tool temperature), *v*_i_ = 20 cm^3^ s^− 1^(injection speed), shot volume of 4.4 cm^3^ and a cooling time (*t*_c_) of 5 s. This resulted in a total cycle time 12 s for each sample.

### 2.3 SEM characterisation

The bare devices were sputtered with 50 nm chromium and imaged using a JEOL 5610 scanning electron microscope.

### 2.4 Mechanical evaluation

#### 2.4.1 Measurement of axial compression force

The effect of applying an axial compression load to the microneedle array was assessed using an Instron 5866 instrument with a 500 N load cell. Microneedle arrays were placed on a fixed metal plate with the microneedles facing upwards before applying the desired force through the movable probe of the Instron compression system. The Instron instrument applied pressure on the microneedle arrays using an axial force (parallel to the microneedles' axes) at a rate of 1 mm/s until the required force was exerted. Forces ranging from 50 N to 400 N were tested. Scanning electron microscopy of microneedle arrays was obtained before and after application of the compression load. The height of each microneedle was measured after testing and the percentage change in microneedle height calculated.

#### 2.4.2 Measurement of transverse fracture force

The transverse failure forces of microneedle arrays were measured with the same force station as described above. A thin probe (Agar Scientific) (2 mm thickness at the tip) was slotted in the clamp and used for this purpose. It was adjusted to ensure that it pressed orthogonally against a row of eight microneedles. The probe was moved at a speed of 1 mm/min. The force required to fracture a single microneedle was determined by dividing the transverse force required to fracture one row by the number of microneedles in each row (four). The microneedle arrays were examined by scanning electron microscopy prior to and after fracture testing.

### 2.5 Functionalisation

Bare microneedle arrays were sputtered with chromium (15 nm)/Platinum (50 nm) to obtain the working electrodes. One of the microneedle arrays was sputtered with Ag (150 nm), which was modified to an Ag/AgCl reference electrode by treating with a saturated solution of FeCl_3_. The working electrodes were biased against the integrated Ag/AgCl reference electrodes for electropolymerisation and subsequent chronoamperometry measurements.

The glucose, lactate and theophylline biosensors described here were fabricated using an electropolymerisation method. For electropolymerised polyphenols, the electrode was placed in 50 mM phenol monomer and 10 mg/mL of GOx/LOx/XOx enzyme dissolved in 100 mM PBS. The pH of the solution was buffered to pH 7.2. Each cycle comprised of holding the working electrode at 0 V for 20 s, polarising it to a potential of 0.9 V for 15 min for electropolymerisation of the film. Each cycle was repeated six times to obtain the desired film thickness and enzyme loading.

Using 200 μL of various concentrations of glucose, lactate and theophylline solutions, chronoamperometric measurements were made over 60 s. From these measurements, dose response curves were obtained for glucose, lactate and theophylline biosensors.

### 2.6 Optical coherence tomography of microneedles in human tissue

Unfunctionalised devices were sterilized by subjecting them to 25 kGy of Co60 (Synergy Health) before the insertion studies. The microneedle array structures were inserted into the forearm of volunteers by the application of moderate thumb pressure (< 10 N) for 60 s.

Optical coherence tomography (details) based has been reportedly used by several groups to look at the skin penetration [5]. On removal of the device, OCT equipment (Vivosight, Michelson diagnostics) was used to look at the penetration of the stratum corneum.

## 3 Results

### 3.1 SEM characterisation

The SEM images were analysed for geometry. Each injection molded part was 20 × 20 mm with a 2 mm thick base and comprising 4 arrays each of which had 16 microneedles in a 4 × 4 subarray. The individual microneedles were pyramidal in shape; 1000 μm tall, with a 600 μm square base and a tip diameter of ~ 40 μm. The pitch between the microneedles is 1200 μm.

### 3.2 Mechanical evaluation

In axial compression tests (*n* = 4) the microneedles tolerated large forces without fracture. The reduction of microneedle height increased non-linearly with increasing applied force. This ranged from 1% for 50 N to 17% for 400 N axial forces. Transverse fracture tests (*n* = 3) showed that the force required to fracture one row of four microneedles was 80 ± 3 N (20 N per microneedle).

### 3.3 Functional evaluation

**Fig. 1:**
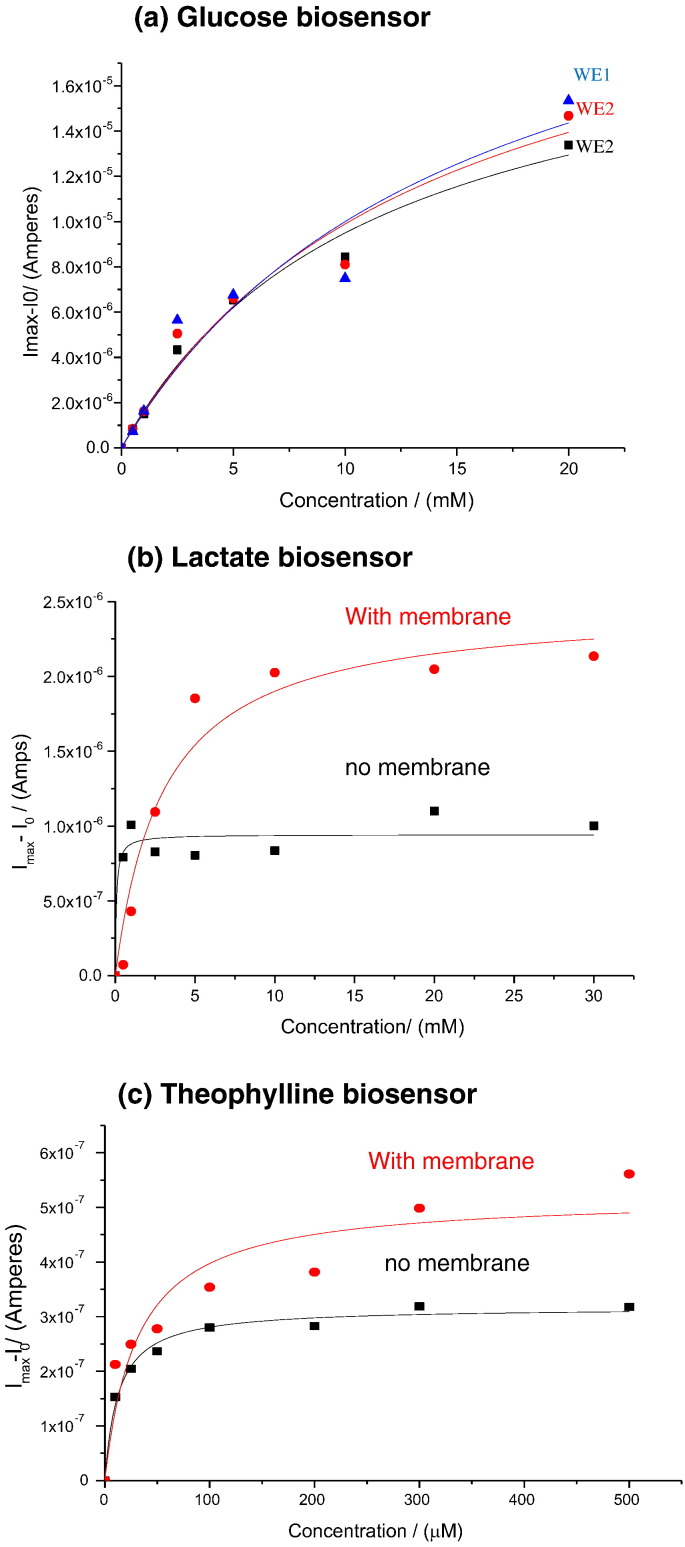
Dose-response curves obtained for glucose, lactate and theophylline. Panel (a) is a dose-response curve obtained from three working electrodes functionalised with glucose oxidase entrapped in polyphenol but without an epoxy PU membrane. In panels (b) and (c) the red curves are for working electrodes coated with epoxy PU membrane and the black curves are for working electrodes without the membrane.

The functionalised devices were tested with varying glucose, lactate and theophylline concentrations using chronoamperometry. From the chronoamperometry studies the dose response curves were fitted to the Michaelis-Menten equation (Fig. 1).

As seen from the dose response curves, glucose and lactate concentration as low as 0.5 mM could be easily detected. For the glucose biosensors, the three microneedle electrode arrays within a single device exhibited Km values of 11.4 ± 2.7 mM, 13.9 ± 5.9 mM and 15.5 ± 9.6 mM. The corresponding I_max_ values for the three electrodes were 20.3 ± 2.4 μA, 23.6 ± 5.4 μA and 25.5 ± 8.7 μA (Table 1).

The I_max_ values were found to be low for the lactate and theophylline biosensors. This can be attributed to the low activity of the enzymes used here (Lactate oxidase 41 U/mg powder and Xanthine oxidase 7 U/mg powder @ 37 °C). With the lactate biosensors, the microneedle array electrodes exhibited Km values of 3.03 ± 0.9 mM with the epoxy polyurethane (PU) membrane and 0.7 ± 0.08 mM respectively. The I_max_ values for the two electrodes were 2.5 ± 0.2 μA and 0.95 ± 0.06 μA respectively. Similarly, the theophylline biosensors exhibited Km values of 31 ± 1.22 μM with the epoxy polyurethane (PU) membrane and 13 ± 1.84 μM respectively. The I_max_ values for the two electrodes were 0.52 ± 0.05 μA and 0.31 ± 0.008 μA respectively.

Whilst the increase in linear range is expected for a mass transport limiting membrane, it would also be expected to reduce the maximum current. A possible explanation for the increased limiting currents is that the membrane reduces the rate of diffusion of hydrogen peroxide away from the electrode surface, resulting in an increased local concentration quickly consequently leading to a higher current.

### 3.4 Insertion test on human skin

**Fig. 2:**
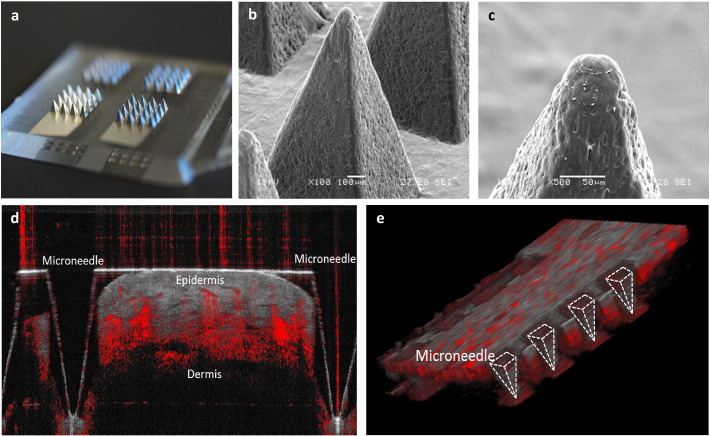
(a) Showing image of the polycarbonate microneedle array sputtered with 50 nm platinum. (b) & (c) Scanning electrochemical microscope images of the microneedle at 100 × and 500 × magnification. (d) Optical coherence tomographic image showing the section of tissue with one embedded microneedle as seen here the microneedle penetrates 800 μm deep into the skin. (e) Graphics produced in ImageJ using the 3D Viewer plugin showing a row of the microneedles embedded in the tissue.

The insertion ratio, as determined from the tests (*n* = 16) *in vivo* (human skin) was 100% for very moderate forces (< 10 N). Whilst the SEM of the microneedle arrays following insertion tests confirmed the structural integrity of the device the OCT images confirm the penetration of the *stratum corneum* (Fig. 2).

It is observed that the channels created by penetration of microneedles collapse on account of the highly elastic nature of the skin layers therefore OCT was done with device inserted.

## 4 Conclusions

Next generation wearable devices will carry minimally invasive, continuous monitoring systems for metabolites, biomarkers and drugs and will have implications for the better management of both lifestyle and chronic diseases. We have demonstrated here a scalable (300/hour) and cost effective approach to fabricating polycarbonate-based (material cost of 2 pence) microneedle structures with desired geometry and mechanical properties. The sterilized structures were tested on human skin and it was observed that the geometry of the tips allowed penetration of the *stratum corneum* and access to the dermal interstitial fluid by insertion under moderate thumb pressure. The microneedle arrays could subsequently be functionalised to perform as electrochemical biosensors.

## Figures and Tables

**Table 1:** Showing the Michaelis-Menten K_M_ constant and the maximum limiting current I_max_ for the three biosensors.

Biosensor	Enzyme	Km	I_max_
Theophylline biosensor	Xanthine oxidase (7 U/mg)		
With epoxy PU membrane		31 μM ± 1.22	0.52 μA ± 0.05
Without membrane		13 μM ± 1.84	0.31 μA ± 0.008
Lactate biosensor	Lactate oxidase (41 U/mg)		
With epoxy PU membrane		3.03 mM ± 0.9	2.5 μA ± 0.2
Without membrane		0.7 mM ± 0.08	0.95 μA ± 0.06
Glucose biosensor	Glucose oxidase (239 U/mg)		
WE1 no membrane		11.4 mM ± 2.74	20.3 μA ± 2.45
WE2 no membrane		13.9 mM ± 5.9	23.6 μA ± 5.4
WE3 no membrane		15.5 mM ± 9.6	25.5 μA ± 8.71
